# Monoterpenoids from *Acanthopanax sessiliflorus* Fruits

**DOI:** 10.3390/molecules18033043

**Published:** 2013-03-07

**Authors:** Chun-Juan Yang, Zhi-Bin Wang, Pu-Yuan Song, Yang Xiao, Yong-Hai Meng, Yan-Yan Wang, Hai Jiang, Hai-Xue Kuang

**Affiliations:** 1 College of Pharmacy, Harbin Medical University, No. 157 Baojian Road, Nangang Distrct, Harbin 150081, China; E-Mail: chunjuanyang@126.com (C.-J.Y.); 2 Key Laboratory of Chinese Materia Medica (Ministry of Education), Heilongjiang University of Chinese Medicine, Harbin 150040, China; E-Mail: 15845002546@139.com (Y.-H.M.)

**Keywords:** *Acanthopanax sessiliflorus* fruits, Araliaceae, monoterpenoids

## Abstract

Three new acyclic monoterpenoids named (2*E*)-3,7-dimethylocta-2,6-dienoate-6-*O*-*α*-l-arabinopyranosyl-(1→6)-*β*-d-glucopyranoside (**1**), (3*Z*,6*E*)-3,7-dimethyl-3,6-octadiene-1,2,8-triol (**2**) and (6*E*)-7-methyl-3-methylene-6-octene-1,2,8-triol (**3**) were isolated from *Acanthopanax sessiliflorus* fruits, along with three known monoterpenoid compounds. The structures of the new compounds were determined by means of extensive spectroscopic analysis (1D, 2D NMR and HRESIMS) and chemical methods.

## 1. Introduction

**Figure 1:**
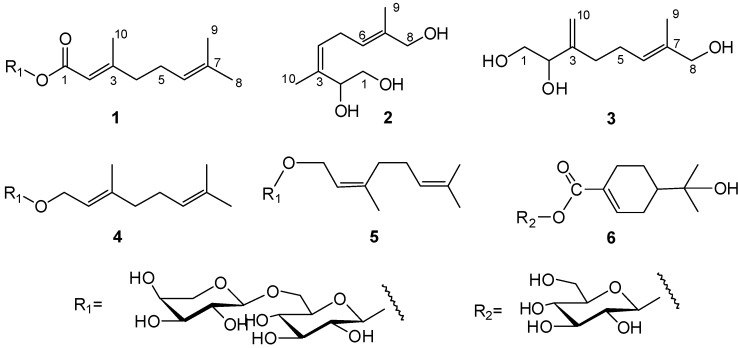
Structures of compounds **1**–**6**.

*Acanthopanax* species (Araliaceae) are shrubs found mainly in China, Korea, and Japan. *Acanthopanax sessiliflorus* (Rupr. et Maxim) Seem. is known to be one of the most abundant species, and the pharmacology research has demonstrated that the fruits of *Acanthopanax* have many biological functions, including antitumor [1,2], immunostimulating [2], antithrombosis [3], antiplatelet activities [4,5], pancreatic lipase-inhibiting properties [6] and effects on lipopolysaccharide-induced nitric oxide production [7]. To further investigate the constituents and screen the bioactive compounds from *A*. *sessiliflorus* fruits, a phytochemical study was performed. From the ethanolic extract of *A.* *sessiliflorus* fruits, three new monoterpenes, (2*E*)-3,7-dimethylocta-2,6-dienoate-6-*O*-*α*-l-arabinopyranosyl-(1→6)-*β*-d-glucopyranoside (**1**), (3*Z*,6*E*)-3,7-dimethyl-3,6-octadiene-1,2,8-triol (**2**) and (6*E*)-7-methyl-3-methylene-6-octene-1,2,8-triol (**3**), were isolated and identified along with the known compounds kenposide A (**4**) [8], sacranoside B (**5**) [9] and 1-*O*-[(*S*)-oleuropeyl]-*β*-d-glucopyranose (**6**) [10], on the basis of spectral analysis, including MS, ^1^H-NMR, ^13^C-NMR, DEPT, HMBC, HMQC and NOESY experiments (Figure 1). In the present report, we describe the structural elucidation of compounds **1**~**3**.

## 2. Results and Discussion

Compound **1** was obtained as a colorless amorphous powder. ${\left[\alpha \right]}_{\text{D}}^{25}$ −31.0 (*c* 0.3, MeOH); UV max (MeOH): 221 (log *ε* 3.95) nm. The IR spectrum of compound **1** showed absorption bands at 3410, 1715, 1645, 1423, 1385, and 1073 cm^−1^ assignable to hydroxyl, ester carbonyl, olefin and ether functions. The molecular formula C_21_H_34_O_11_ was defined by HRESIMS (*m/z*: 485.1995 [*M*+Na]^+^, calc. for C_21_H_34_O_11_Na 485.1999). Acid hydrolysis of **1** liberated d-glucose and l-arabinose, which were identified by GC analysis using a hydrogen flame detector after treatment with l-cysteine methyl ester hydrochloride in pyridine [4,11].

^1^H-NMR and ^13^C-NMR spectra of **1** (Table 1) showed the presence of three vinyl methyls [*δ* 1.68 (s, 3H, H-8)], [*δ* 1.62 (s, 3H, H-9)], [*δ* 2.18 (s, 3H, H-10)], two trisubstituted alkenes [*δ* 5.10 (dd, 1H, *J* = 6.6, 6.9 Hz, H-6), *δ* 5.74 (s, 1H, H-2)], and two anomeric protons [*δ* 5.47 (d, 1H, *J* = 8.2 Hz, H-1')], [*δ* 4.27 (d, 1H, *J* = 6.2 Hz, H-1'')]. ^1^H-^1^H correlations were observed between the following protons pairs: *δ* 2.20 (H-4) and *δ* 2.21 (H-5); *δ* 2.21 (H-5) and *δ* 5.10 (H-6), suggesting a –CH_2_CH_2_CH– fragment. Two anomeric proton signals were assigned to two anomeric carbon signals at *δ* 95.2 (C-1') and 104.8 (C-1'') respectively, in the HMQC experiment. By comparing coupling constants and the chemical shifts of the sugar signals with those reported [12,13], the two sugars were deduced to be of *β*-configuration for glucose and *α* for arabinose. In the HMBC spectrum, long-range correlations between the methyl proton signals at *δ* 2.18 (H-10) and the carbon signals at *δ* 115.9 (C-2), 164.4 (C-3) and 42.0 (C-4) could be identified. Moreover, the long-range correlations were observed between the following protons and carbons: *δ* 5.74 (H-2) and *δ* 166.5 (C-1), 164.4 (C-3), 42.0 (C-4), 19.3 (C-10); *δ* 2.21 (H-5) and *δ* 42.0 (C-4), 124.1 (C-6), 133.6 (C-7); *δ* 1.62 (H-9) and *δ* 124.1 (C-6), 133.6 (C-7) , 25.9 (C-8); *δ* 5.47 (H-1') and *δ* 166.5 (C-1); *δ* 4.27 (H-1'') and *δ* 69.2 (C-6'). In the NOESY spectrum, NoE correlations were observed between the following protons and protons: *δ* 5.74 (H-2) and *δ* 2.20 (H-4), which indicated a trisubstituted alkene with the *trans*-configuration in the 2 position. Thus, the structure of compound **1** could be elucidated as (2*E*)-3,7-dimethylocta-2,6-dienoate-6-*O*-*α*-l-arabinopyranosyl-(1→6)-*β*-d-glucopyranoside.

**Table 1:** ^1^H- and ^13^C-NMR data for **1** (600 and 150 MHz, CD_3_OD, *J* in Hertz and *δ* in ppm).

Position	*δ*_H_	*δ*_C_	HMBC (from H to C)	NOESY
**1**		166.5		
**2**	5.74 (s, 1H)	115.9	C-1, C-3, C-4, C-10	H-4
**3**		164.4		
**4**	2.20 (m, 2H)	42.0	C-2, C-3, C-5, C-6, C-10	H-2
**5**	2.21 (m, 2H)	27.1	C-3, C-4, C-6, C-7	H-9
**6**	5.10 (dd, 1H, *J* = 6.6, 6.9)	124.1	C-4, C-5, C-8, C-9	H-8
**7**		133.6		
**8**	1.68 (s, 3H)	25.9	C-6, C-7, C-9	H-6
**9**	1.62 (s, 3H)	17.8	C-6, C-7, C-8	H-5
**10**	2.18 (s, 3H)	19.3	C-2, C-3, C-4	
**1'**	5.47 (d, 1H, *J* = 8.2)	95.2	C-1, C-5'	
**2'**	3.54 (overlapped)	77.9		
**3'**	3.43 (dd, 1H, *J* = 8.9, 8.9)	77.7		
**4'**	3.52 (overlapped)	73.9		
**5'**	3.39 (m, 1H)	71.2		
**6'**	3.72 (dd, 1H, *J* = 5.5, 11.0)	69.2		
	4.10 (dd, 1H, *J* = 2.1, 11.0)			
**1''**	4.27 (d, 1H, *J* = 6.2)	104.8	C-6', C-5''	
**2''**	3.57 (dd, 1H, *J* = 6.2, 8.2)	72.4		
**3''**	3.34 (dd, 1H, *J* = 8.2, 8.9)	74.2		
**4''**	3.78 (m, 1H)	69.6		
**5''**	3.51 (dd, 1H, *J* = 5.5, 12.4)	66.7		
	3.85 (dd, 1H, *J* = 2.8, 12.4)			

Compound **2** was obtained as a yellow oil. ${\left[\alpha \right]}_{\text{D}}^{25}$ +15.0 (*c* 0.3, MeOH); The molecular formula C_10_H_18_O_3_ was defined by HRESIMS (*m/z*: 209.1153 [*M*+Na]^+^, calc. for C_10_H_18_O_3_Na 209.1154). The ^1^H-NMR and ^13^C-NMR spectra of **2** (Table 2) showed the presence of two methyls [*δ* 1.68 (s, 3H, H-10), 1.67 (s, 3H, H-9)], two methylenes bearing an oxygen function [*δ* 3.55 (dd, 1H, *J* = 11.0, 8.3 Hz, H-1a), 3.46 (dd, 1H, *J* = 11.0, 5.5 Hz, H-1b), 3.91 (s, 2H, H-8)], a methylene [*δ* 2.87 (m 1H, H-5a), 2.79 (m 1H, H-5b), two alkene methines [*δ* 5.29 (dd, 1H, *J* = 7.6, 6.8 Hz, H-4), 5.38 (dd, 1H, *J* = 7.6, 6.8 Hz, H-6)] and a methine [*δ* 4.61 (dd, 1H, *J* = 8.3, 5.5, H-2)].

**Figure 2:**
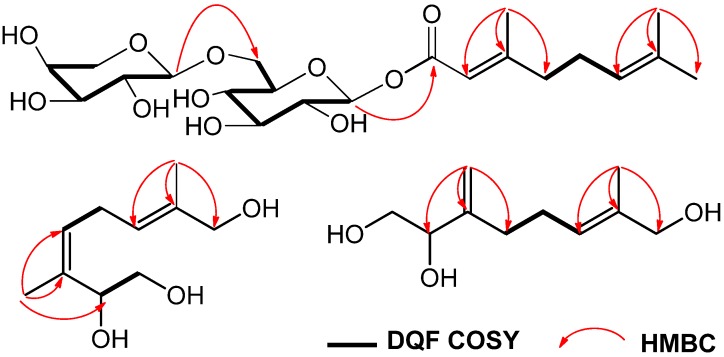
Key HMBC and ^1^H-^1^H COSY correlations of **1**–**3**.

The ^1^H-^1^H COSY spectrum showed correlations between *δ* 3.46 (H-1a), 3.55 (H-1b) and *δ* 4.61 (H-2), suggesting a –CHOHCH_2_OH fragment. ^1^H-^1^H correlations were observed between the following proton pairs: *δ* 5.29 (H-4) and *δ* 2.87 (H-5a); *δ* 2.87 (H-5a) and *δ* 5.38 (H-6), suggesting a –CHCH_2_CH– fragment. Moreover, in the HMBC spectrum, long-range correlations between the methyl proton signals at *δ* 1.68 (H-10) and the carbon signals at *δ* 71.6 (C-2), 136.3 (C-3) and 127.6 (C-4) could be identified. Correlations were also observed between the methyl proton signals at *δ* 1.67 (H-9) and the carbon signals at *δ* 125.2 (C-6), 136.2 (C-7) and 68.9 (C-8) (Figure 2).

**Table 2:** ^1^H- and ^13^C-NMR data for **2** and **3** (600 and 150 MHz, CD_3_OD, *J* in Hertz and *δ* in ppm).

Position	2		3	
	*δ*_H_	*δ*_C_	*δ*_H_	*δ*_C_
1	3.46 (1H, dd, *J* = 11.0, 5.5)	65.6	3.59 (1H, dd, *J* = 11.0, 3.7)	66.5
	3.55 (1H, dd, *J* = 11.0, 8.3)		3.47 (1H, dd, *J* = 11.0, 7.0)	
2	4.61 (1H, dd, *J* = 8.3, 5.5)	71.6	4.09 (1H, dd, *J* = 7.0, 3.7)	76.6
3		136.3		150.3
4	5.29 (1H, dd, *J* = 7.6, 6.8)	127.6	2.07 (1H, m), 2.16 (1H, m)	33.1
5	2.87 (1H, m), 2.79 (1H, m)	27.0	2.24 (2H, m)	27.2
6	5.38 (1H, dd, *J* = 7.6, 6.8)	125.2	5.43 (1H, dd, *J* = 6.8, 6.9)	126.3
7		136.2		136.3
8	3.91 (2H, s)	68.9	3.91 (2H, s)	68.9
9	1.67 (3H, s)	13.8	1.66 (3H, s)	13.8
10	1.68 (3H, s)	18.5	4.93 (1H, d, *J* = 1.4)	111.3
			5.11 (1H, d, *J* = 1.4)	

In the NOESY spectrum, NOE correlations were observed between the following proton pairs: *δ* 1.68 (H-10) and *δ* 5.29 (H-4); *δ* 5.38 (H-6) and *δ* 3.91 (H-8), which indicated a trisubstituted alkene with the *cis*-configuration in the 3 position and another alkene with the *trans*-configuration in the 6 position. Thus, the structure of compound **2** could be elucidated as (3*Z*,6*E*)-3,7-dimethyl-3,6-octadiene-1,2,8-triol.

Compound **3** was isolated as a yellow oil. ${\left[\alpha \right]}_{\text{D}}^{25}$+30.0 (*c* 0.3, MeOH). The molecular formula C_10_H_18_O_3_ was defined by HRESIMS (*m/z*: 209.1150 [*M*+Na]^+^, calc. for C_10_H_18_O_3_Na 209.1154). The NMR data (Table 2) showed an alkene methylene [*δ* 4.93 (1H, d, *J* = 1.4 Hz, H-10a), 5.11(1H, d, *J* = 1.4 Hz, H-10b)], a methyl [*δ* 1.66 (3H, s, H-9)] and a methine [*δ* 5.43 (dd, 1H, *J* = 6.8, 6.9 Hz, H-6)]. The structure of **3** was subsequently assigned by HMQC, DQF COSY and HMBC. In the HMBC spectrum (Figure 2), the long-range correlations were observed between the following protons and carbons: *δ* 4.93 (H-10a) and *δ* 76.6 (C-2), 150.3 (C-3), 33.1 (C-4); *δ* 1.66 (H-9) and *δ* 126.3 (C-6), 136.3 (C-7), 68.9 (C-8). Finally, the configuration of the trisubstituted alkene was clarified by a difference NOE experiment. The irradiation of *δ* 3.91 (H-8) resulted in an enhancement at *δ* 5.43 (H-6), suggesting the *trans*-configuration at the 6 position, Therefore, compound **3** was identified as (6*E*)-7-methyl-3-methylene-6-octene-1,2,8-triol.

## 3. Experimental

### 3.1. General

Open column chromatography was carried out using silica gel (200–300 mesh, Qingdao Marine Chemical Co., Qingdao, China) or octadecyl silica gel (ODS, 25–40 μm, Fuji, Tokyo, Japan) as stationary phase. TLC employed precoated silica gel plates (5–7 μm, Qingdao Marine). Preparative HPLC was carried out on a Waters 600 instrument equipped with a Waters RID-2414 detector. A Waters Sunfire prep C18 OBD (19 × 250 mm i.d.; Waters, Milford, MA, USA) column was used for preparative purpose. The IR spectra were recorded as KBr pellets on a Jasco 302-A spectrometer (Jasco, Tokyo, Japan). The UV spectra were recorded on a Shimadzu UV-2450 spectrophotometer (Shimadzu, Kyoto, Japan). Optical rotation was recorded on a Jasco P-2000 polarimeter. HRESIMS were measured on a FTMS-7 instrument (Bruker Daltonics, Karlsruhe, Germany). The ^1^H, ^13^C and 2D (^1^H-^1^H COSY, HMQC, HMBC, NOESY) NMR spectra were recorded on a JNM-ECA600 spectrometer (JEOL, Tokyo, Japan) using standard pulse sequence. Chemical shifts were reported in ppm (*δ*), and scalar coupling were reported in Hz. GC analyses were carried out using a Fuli 9790 instrument, DM-5 column (0.25 μm, 30 m × 0.25 mm, Dikma, China).

### 3.2. Plant

The fruits of *A. sessiliflorus* (Rupr. et Maxim.) Seem. were collected from WuJia Agriculture Sci-Tech Co. Ltd. of Dandong, Liaoning Province, China in October, 2009 and identified by Professor Jincai Lu of School of Traditional Chinese Materia Medica of Shenyang Pharmaceutical University, China.

### 3.3. Extraction and Isolation

The dried and powdered fruits (12 kg) of *A*. *sessiliflorus* (Rupr. et Maxim.) Seem. were extracted with 70% EtOH (3 × 32 L) under reflux (1 h). The combined extract was concentrated under vacuum yielding a residue (2.2 kg) which was dissolved in water, loaded on a D101 macroporous adsorption resin column and eluted successively with H_2_O, 30% EtOH, 60% EtOH and 95% EtOH.

The 30% EtOH fraction (100.0 g) was subjected to silica gel column chromatography with a stepwise gradient CHCl_3_–MeOH (10:1, 5:1, 3:1, 2:1, 1:1 *v/v*), and finally with MeOH alone, to give five fractions 1–6. Fraction 4 (9.7 g) was subjected to reversed-phase silica gel column chromatography [200 g, MeOH–H_2_O (10:90→20:80→30:70→40:60→50:50, *v/v*)→MeOH] to afford six fractions [Fr. 4.1 (3.0 g), Fr. 4.2 (506 mg), Fr. 4.3 (755 mg), Fr. 4.4 (321 mg), Fr. 4.5 (612 mg), Fr. 4.6 (538 mg)]. Fr. 4.2 (506 mg) was separated by HPLC [MeCN–H_2_O (7: 93, *v/v)*] to give **2** (10.6 mg) and **3** (11.2 mg). Fraction 4.3 (755 mg) was separated by HPLC [MeOH–H_2_O (20:80, *v/v*)] to give **6** (15.8 mg). Fraction 5 (15.1 g) was subjected to reversed-phase silica gel column chromatography [300 g, MeOH–H_2_O (10:90→20:80→30:70→50:50→60:40, *v/v*)→MeOH] to afford ten fractions [Fr. 5.1 (1.45 g), Fr. 5.2 (1.61 g), Fr. 5.3 (168 mg), Fr. 5.4 (995 mg), Fr. 5.5 (217 mg), Fr. 5.6 (1.1 g), Fr. 5.7 (121 mg), Fr. 5.8 (674 mg), Fr. 5.9 (2.0 g), Fr. 5–10 (197 mg)]. Fraction 5.2 (533 mg) was separated by HPLC [MeOH–H_2_O (35:65, *v/v*)] to give **1** (20.1 mg). Fraction 5.3 (197 mg) was separated by HPLC [MeOH–H_2_O (35:65, *v/v*)] to give **4** (15.3 mg). Fraction 5.4 (426 mg) was separated by HPLC [MeOH–H_2_O (40:60, *v/v*)] to give **5** (8.5 mg). The known compounds kenposide A (**4**) [8], sacranoside B (**5**) [9] and 1-*O*-[(*S*)-oleuropeyl]-*β*-d-glucopyranose (**6**) [10] were identified by comparison of their analytical data ([α]_D_, ^1^H-NMR, ^13^C-NMR, MS) with those reported.

*(2E)-3,7-Dimethylocta-2,6-dienoate-6-O-α-l-arabinopyranosyl-(1→6)-β-d-glucopyranoside* (**1**). ${\left[\alpha \right]}_{\text{D}}^{25}$ –31.0 (*c* 0.3, MeOH). HR-ESI-MS *m/z* 485.1995 [*M*+Na]^+^ (calc. C_21_H_34_O_11_Na, 485.1999); UV max (MeOH) 221 nm; IR (KBr) 3410, 1715, 1645, 1423, 1385, and 1073 cm^−1^; ^1^H- and ^13^C-NMR (CD_3_OD) data, see Table 1.

*(3Z,6E)-3,7-Dimethyl-3,6-octadiene-1,2,8-triol* (**2**). ${\left[\alpha \right]}_{\text{D}}^{25}$ +15.0 (*c* 0.3, MeOH). HR-ESI-MS *m/z* 209.1153 [*M*+Na]^+^ (calc. C_10_H_18_O_3_Na, 209.1154); UV max (MeOH): 205 nm; IR (KBr) 3355, 2971, 1717 and 1160 cm^−1^; ^1^H- and ^13^C-NMR (CD_3_OD) data, see Table 2.

*(6E)-7-Methyl-3-methylene-6-octene-1,2,8-triol* (**3**). ${\left[\alpha \right]}_{\text{D}}^{25}$ +30.0 (*c* 0.3, MeOH). HR-ESI-MS *m/z* 209.1150 [*M*+Na]^+^ (calc. C_10_H_18_O_3_Na, 209.1154); UV max (MeOH): 202 nm; IR(KBr) 3903, 2965, 1668 and 1055 cm^−1^; ^1^H- and ^13^C-NMR (CD_3_OD) data, see Table 2.

### 3.4. Acid Hydrolysis of and Determination of the Absolute Configuration of the Monosaccharides

Compound **1** (3.03 mg) was hydrolyzed with 1 M HCl (1.0 mL) for 2 h at 85 °C. The reaction mixture was cooled and partitioned between CHCl_3_ (2.0 mL) and H_2_O (2.0 mL). The aqueous layer was washed with CHCl_3_ (3.0 mL × 3), neutralized with Ba(OH)_2_, filtered, and evaporated under reduced pressure. The residue was dissolved in pyridine (1.0 mL) and 0.1 M L-cysteine methyl ester hydrochloride in pyridine (2.0 mL) was added. The mixture was heated at 60 °C for 1 h. An equal volume of Ac_2_O was added with heating continued 1 h. The acetylated thiazolidine derivatives were analyzed by GC using a DM-5 Column (30 m × 0.25 mm, 0.25 μm). Temperatures of injector and detector were 280 °C for both. A temperature gradient system was used for the oven; starting at 160 °C and increasing up to 195 °C at a rate of 5 °C/min. Peaks of the hydrolysate were detected by comparison with retention time of authentic samples of d-glucose (10.08 min) and l-arabinose (6.55 min) after treatment with l-cysteine methyl ester hydrochloride in pyridine.

## 4. Conclusions

In this paper, three new acyclic monoterpenoids, (2*E*)-3,7-dimethylocta-2,6-dienoate-6-*O*-*α*-l-arabinopyranosyl-(1→6)-*β*-d-glucopyranoside (**1**), (3*Z*,6*E*)-3,7-dimethyl-3,6-octadiene-1,2,8-triol (**2**) and (6*E*)-7-methyl-3-methylene-6-octene-1,2,8-triol (**3**) were isolated from the EtOH extract of the dried fruits of *Acanthopanax sessiliflorus* together with three known monoterpenoids. To the best of our knowledge, this is the first scientific report of acyclic monoterpenoids from *Acanthopanax* plants.

## References

[1] Lee D.Y., Seo K.H., Lee D.S., Kim Y.C., Chung I.S., Kim G.W., Cheoi D.S., Baek N.I. (2012). Bioactive 3,4-seco-triterpenoids from the fruits of *Acanthopanax sessiliflorus*. J. Nat. Prod..

[2] Lee S.H., Lee Y.S., Jung S.H., Ji J., Shin K.H., Kin B.K., Kang S.S. (2003). Antitumor and immunostimulating activities of *Acanthopanax sessiliflorus* fruits. Nat. Prod. Sci..

[3] Song Y., Yang C.J., Yu K., Li F.M. (2011). In vivo antithrombotic and antiplatelet activities of a quantified *Acanthopanax sessiliflorus* fruit extract. Chin. J. Nat. Med..

[4] Yang C.J., An Q., Xiong Z.L., Song Y., Yu K., Li F.M. (2009). Triterpenes from *Acanthopanax sessiliflorus* fruits and their antiplatelet aggregation activities. Planta Med..

[5] Jin J.L., Lee S., Lee Y.Y., Kim J.M., Heo J.E., Yun-Choi H.S. (2004). Platelet anti-aggregating triterpenoids from the leaves of *Acanthopanax senticosus* and the fruits of *A. sessiliflorus*. Planta Med..

[6] Li F., Li W., Fu H., Zhang Q., Koike K. (2007). Pancreatic lipase-inhibiting triterpenoid saponins from fruits of *Acanthopanax senticosus*. Chem. Pharm. Bull..

[7] Yang E.J., Moon J.Y., Lee J.S., Koh J., Lee N.H., Hyun C.G. (2010). *Acanthopanax koreanum* fruit waste inhibits lipopolysaccharide-induced production of nitric oxide and prostaglandin E2 in RAW 264.7 macrophages. J. Biomed. Biotechnol..

[8] Yoshikawa M., Shimada H., Horikawa S., Murakami T., Shimoda H., Yamahara J., Matsuda H. (1997). Bioactive constituents of Chinese natural medicines. IV. Rhodiolae radix. (2).: On the histamine release inhibitors from the underground part of *Rhodiola sacra* (Prain ex Hamet) S. H. Fu (Crassulaceae): Chemical structures of rhodiocyanoside D and sacranosides A and B. Chem. Pharm. Bull..

[9] Yu Y., Gao H., Dai Y., Wang Y., Chen H.R., Yao X.S. (2010). Monoterpenoids from the fruit of *Gardenia jasminoides*. Helv. Chim. Acta.

[10] Goodger J.Q., Cao B., Jayadi I., Williams S.J., Woodrow I.E. (2009). Non-volatile components of the essential oil secretory cavities of *Eucalyptus leaves*: Discovery of two glucose monoterpene esters, cuniloside B and froggattiside A. Phytochemistry.

[11] Yang C.J., Wang Z.B., Zhu D.L., Yu Y., Lei Y.T., Liu Y. (2012). Two new cyanogenic glucosides from the leaves of *Hydrangea macrophylla*. Molecules.

[12] Wang Z.B., Gao H.Y., Xu F.M., Wu L.J. (2010). Three new compounds from the leaves of *Acanthopanax senticosus* harms. Chin. Chem. Lett..

[13] Nakamura S., Li X.Z., Matsuda H., Yoshikawa M. (2008). Bioactive constituents from Chinese natural medicines. XXVIII. Chemical structures of acyclic alcohol glycosides from the roots of *Rhodiola crenulata*. Chem. Pharm. Bull..

